# A retrospective analysis of cross-reacting cetuximab IgE antibody and its association with severe infusion reactions

**DOI:** 10.1002/cam4.333

**Published:** 2014-10-09

**Authors:** Sabine Maier, Christine H Chung, Michael Morse, Thomas Platts-Mills, Leigh Townes, Pralay Mukhopadhyay, Prabhu Bhagavatheeswaran, Jan Racenberg, Ovidiu C Trifan

**Affiliations:** 1 Bristol-Myers Squibb CompanyLawrenceville, New Jersey; 2 Johns Hopkins UniversityBaltimore, Maryland; 3 Duke University Medical CenterDurham, North Carolina; 4 University of VirginiaCharlottesville, Virginia; 5 Bristol-Myers Squibb CompanyWallingford, Connecticut

**Keywords:** Biomarkers, cetuximab, colorectal neoplasms, head and neck neoplasms, immunoglobulin E, lung neoplasms

## Abstract

Severe infusion reactions (SIRs) at rates of 5% or less are known side effects of biological agents, including mAbs such as cetuximab. There are currently no prospectively validated risk factors to aid physicians in identifying patients who may be at risk of experiencing an SIR following administration of any of these drugs. A retrospective analysis of 545 banked serum or plasma samples from cancer patients participating in clinical trials of cetuximab was designed to evaluate whether the presence of pretreatment IgE antibodies against cetuximab, as determined by a commercially available assay system, is associated with SIRs during the initial cetuximab infusion. Patients with a positive test indicating the presence of pretreatment antibodies had a higher risk of experiencing an SIR; however, at the prespecified cutoff utilized in this analysis, the test has a relatively low-positive predictive value (0.577 [0.369–0.766]) and a negative predictive value of 0.961 (0.912–0.987) in an unselected patient population. Data collected in this large retrospective validation study support prior observations of an association between the presence of pretreatment IgE antibodies cross-reactive with cetuximab and SIRs. Further analysis of the test's ability to predict patients at risk of an SIR would be required before this assay could be used reliably in this patient population.

## Introduction

Infusion reaction is a known side effect of monoclonal antibodies (mAbs) such as trastuzumab, rituximab, bevacizumab, infliximab, cetuximab, and panitumumab. Current labels 1–6 indicate that mild-to-moderate reactions occur in 3–40% of patients with severe infusion reactions (SIRs) occurring in ≤5%. There are no known prospectively validated predictive factors for experiencing an SIR following administration of any of these drugs.

Drug-induced infusion reactions are systemic hypersensitivity reactions (HSRs). HSRs are classified based on the mechanisms involved and the time to induce the reaction 7. Type I HSRs, immediate or anaphylactic reactions, are typically mediated by IgE, which binds to its receptor on basophils and mast cells, releasing immune mediators that evoke a multi-organ systemic response. Type II and III HSRs are mediated by IgG antibodies and the formation of immune complexes. Type IV HSRs are mediated by T cells. In addition to HSRs mediated through specific recognition of the antigen by the immune system, nonimmune-mediated pseudoallergic reactions, which resemble immune system-mediated reactions, are commonly seen with mAbs.

The current cetuximab label states that SIRs (National Cancer Institute Common Toxicity Criteria Grades 3 and 4) occurred in 2–5% of 1373 patients receiving cetuximab in registrational clinical trials, with a fatal outcome in one patient 5. SIRs requiring medical intervention and discontinuation were associated with rapid onset of airway obstruction, hypotension, shock, loss of consciousness, myocardial infarction and/or cardiac arrest (anaphylaxis or infusion-related reaction in the current Common Terminology Criteria for Adverse Events) 5. Approximately 90% of cetuximab-induced SIRs were associated with the first infusion and occurred despite the use of prophylactic antihistamines. SIRs generally developed within 1 h after the initial infusion, but also occurred after several hours or with subsequent infusions.

Safety monitoring in ongoing cetuximab trials and postmarketing pharmacovigilance reports support the 2–5% rate of SIRs reported in the current labeling. However, a few retrospective case series suggested a higher prevalence of SIRs in a southeastern area of the United States (U.S.) 8–10.

The acuteness and severity of symptoms associated with cetuximab-induced SIRs suggested a Type I reaction mediated by preexisting IgE antibodies cross-reactive with cetuximab. A potential association between anti-cetuximab IgE and SIR was first investigated in a retrospective analysis that examined pretreatment serum samples from 76 patients treated with cetuximab 11. Patients were enrolled primarily in Tennessee, Arkansas, and North Carolina—a geographic area with a seemingly higher incidence of SIRs following cetuximab administration 8–10. Twenty-five patients had SIRs, and 17 had IgE antibodies cross-reactive with cetuximab in their pretreatment samples. One of 51 patients who did not experience an SIR had IgE antibodies cross-reactive with cetuximab (*P* < 0.001). Although correlation of pretreatment IgE cross-reactive with cetuximab with SIRs does not prove causation, these results support the hypothesis that preexisting IgE antibodies cross-reactive with cetuximab may be a potential risk factor for severe IgE-mediated Type I HSR.

Galactose-*α*-1,3 galactose (alpha-gal), present on both Fab segments of cetuximab, was identified as the critical epitope that cross-reacts with the preexisting IgE antibodies 11. No other epitopes have been identified. Alpha-gal is a carbohydrate commonly expressed on nonprimate mammalian proteins. The reasons for the presence of IgE antibodies binding alpha-gal in some individuals are not well understood. In 2011, tick bites were described as a possible cause of IgE antibody responses to alpha-gal 12. A recent report described a cohort of patients with IgE antibodies to alpha-gal who experienced delayed symptoms of anaphylaxis, angioedema, or urticaria after eating mammalian meat 13.

We present the results of a retrospective matched-control and cohort evaluation of cancer patients participating in clinical trials of cetuximab, designed to (1) evaluate whether the presence of pretreatment IgE antibodies against cetuximab is associated with SIR during initial infusion and (2) evaluate the positive predictive value (PPV), negative predictive value (NPV), sensitivity, and specificity of the Phadia ImmunoCAP Specific IgE System, which is designed to detect anti-cetuximab IgE using ImmunoCAP Allergen c360, Cetuximab. The ImmunoCAP Specific IgE System was developed by Phadia as an in vitro quantitative assay for the measurement of allergen-specific IgE in human serum or plasma 14,15. This large retrospective validation study highlights the potential of biomarkers to personalize the management of treatment toxicity. The results suggest that quantitative measure of anti-cetuximab IgE rather than simply its presence or absence may be important, and thus highlight the complexity of designing the most appropriate assay to enable accurate prediction of those patients most at risk for an HSR.

## Methods

The primary objective was to assess the association between occurrence of SIRs in patients receiving their initial infusion of cetuximab and presence of pretreatment IgE antibodies cross-reacting with cetuximab. Additional objectives included determining the sensitivity and specificity of the Phadia ImmunoCAP Allergen c360 Cetuximab test.

The study was conducted in accordance with the Declaration of Helsinki and the International Conference on Harmonisation, Good Clinical Practices, Good Epidemiology Practices, and applicable regulatory requirements. Protocols and informed consent forms from the original sponsored clinical trials included in this retrospective analysis were approved by investigators' institutional review boards before each study was initiated and were reviewed to confirm that language was included allowing the retrospective use of clinical data as well as testing of serum, plasma, or whole blood samples for IgE antibodies cross-reactive with cetuximab. Written informed consent was obtained from all patients prior to any tests or evaluations in the original trials.

### Study design and treatment

**Figure 1:**
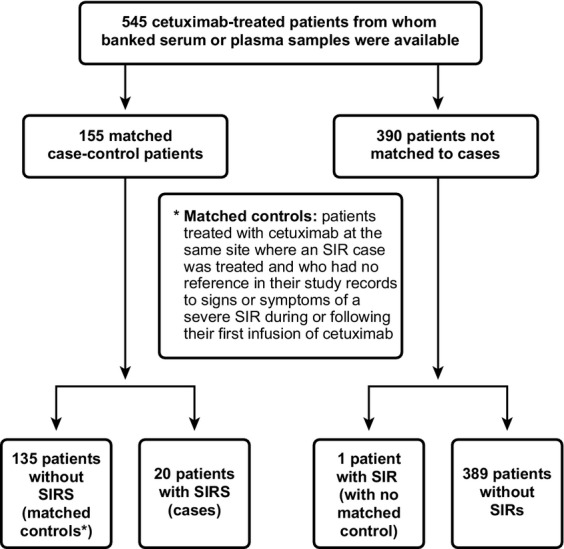
Study schema showing the 545 cetuximab-treated patients from whom banked serum or plasma samples were available. SIR, severe infusion reaction.

This was a retrospective case–control analysis of serum or plasma samples from 19 previously conducted Bristol-Myers Squibb/ImClone–sponsored cetuximab clinical trials (Table1); samples were obtained before administration of cetuximab. The study schema is provided in Figure1.

**Table 1:** Cetuximab studies on which patients included in the current IgE analysis were enrolled.

	Indication	Number of patients receiving cetuximab	Number of samples available
Bristol-Myers Squibb studies			
CA225-004	Advanced cancer	40	35
CA225-005	Advanced cancer	39	21
CA225-006	Colorectal cancer	638	20
CA225-014	Colorectal cancer	50	6
CA225-046	Ovarian cancer	25	4
CA225-058	Non-small cell lung cancer	165	2
CA225-081	Non-small cell lung cancer	57	10
CA225-099	Non-small cell lung cancer	325	8
CA225-100	Non-small cell lung cancer	64	2
ImClone studies			
CP02-0038	Colorectal cancer	30	23
CP02-0141	Colorectal cancer	57	34
CP02-0144	Colorectal cancer	346	282
CP02-9813	Head and neck cancer	21	3
CP02-9814	Pancreatic cancer	41	11
CP02-9815	Head and neck cancer	211	21
CP02-9816	Head and neck cancer	9	9
CP02-9823	Colorectal cancer	46	46
CP02-9925	Non-small cell lung cancer	35	5
CP02-9932	Non-small cell lung cancer	31	3

### Eligibility criteria

Eligible patients included adults who had been previously treated with cetuximab in a clinical trial, with an appropriate sample collected prior to receiving their initial infusion. Patient samples from a previous study 11 were used to develop the ImmunoCAP assay and were therefore excluded from this analysis to allow independent validation. Documentation of the presence or absence of an SIR during the initial infusion of cetuximab was required. Serum/plasma sample volume had to be adequate to obtain a valid readout from the ImmunoCAP Allergen c360 Cetuximab test and to yield an interpretable result for the presence of anti-cetuximab-IgE. Patients who received chimerized or murine mAb therapy before receiving cetuximab were not eligible.

Matching controls consisted of patients who were treated with cetuximab at the same site where a patient who experienced an SIR was treated, but who had no documented reference to the signs or symptoms of an SIR either during or following their initial infusion.

### Assessments

A review of each patient's study records was performed by 2 independent medical reviewers blinded to the test results for IgE antibodies cross-reactive with cetuximab and to each other's assessments. In cases with discordant assessments, a review of the source data by a third blinded medical reviewer was requested, and their assessment was deemed final.

A patient was considered to have an SIR if they met all of the following criteria:

1. Onset of an infusion reaction within 1 h of the initial cetuximab infusion.
2. Presence of at least one sign and/or symptom indicating SIR and described using the terms hypotension, hypoxia, angioedema, unarousable, or unresponsive. Other described signs and symptoms that were clinically consistent with hypotension—hypoxia, angioedema, or unarousable or unresponsive states—were interpreted as indicating these terms, even if the specific term was not reported in the study record.
3. Need for at least 1 parenteral medication to treat the SIR.
4. Discontinuation of further cetuximab treatment per decision of the patient's physician.

Assays for the presence of IgE antibodies cross-reactive with cetuximab were performed using ImmunoCAP Allergen c360, Cetuximab, developed by Phadia. The ImmunoCAP Specific IgE test is based on the principle of a sandwich fluorescence enzyme-linked immunosorbent assay, in which the allergen of interest (e.g., cetuximab) reacts with IgE in a sample taken from a patient. The cetuximab ImmunoCAP does not use streptavidin coating. Cetuximab is covalently coupled to the solid phase, nonspecific IgE is washed away, and enzyme-labeled antibodies against the specific IgE are added to form a complex. After incubation, the unbound enzyme-linked anti-IgE is washed away and a developing agent is incubated with the bound complex. Fluorescence is then measured with higher fluorescence indicative of greater amounts of specific IgE in the original sample. Results are evaluated based on conversion of fluorescence to concentration using a calibration curve.

The limit of quantitation of the ImmunoCAP assay is 0.1 kU_A_/L, and this value was used as the predefined cutoff for the current study. Of note, no prior clinical data were available with the newly developed assay because the prior study used a different assay with a different detection limit (0.35 kU_A_/L) but with same control curve and units and calibrators 11. For a sample to be assigned as IgE positive, the measured amount of IgE antibodies cross-reactive with cetuximab had to be >0.10 kU_A_/L; a sample was categorized as IgE negative if the measured amount was ≤0.10 kU_A_/L. The serum, plasma, or whole blood samples for all patients were analyzed by Phadia personnel who were fully blinded to the patients' clinical data. The detection limit was chosen as cutoff in both the current and prior study, as routinely done for other Phadia ImmunoCAP assays.

### Statistical analyses

To address the primary objective and control for the possible confounding influence of geographic location as observed by O'Neil et al. 8, a matched case–control design with clinical site matching cases to controls was used. For each patient with an SIR identified as a case patient, one or more controls were matched from the same site. A conditional logistic regression model was used to assess the association between the occurrences of SIRs and the presence of IgE antibodies cross-reactive with cetuximab. The resulting odds ratio and its 95% confidence interval (CI), along with the *P* value, were computed.

Secondary objectives were evaluated from all treated patients with IgE samples. Performance characteristics, including sensitivity, specificity, PPV, and NPV, were computed along with 95% CIs using the binomial distribution. The IgE antibodies cross-reactive with cetuximab biomarker status frequencies were summarized by geographic location.

Sensitivity was defined as the proportion of treated patients with an SIR who tested IgE positive. Specificity was defined as the proportion of treated patients without an SIR who tested IgE negative. PPV was defined as the proportion of the total number of IgE-positive patients who had an SIR, whereas NPV was defined as the proportion of the total number of IgE-negative patients who did not have an SIR.

## Results

### Patient disposition

Serum or plasma samples from 545 patients were analyzed for pretreatment IgE antibodies cross-reactive with cetuximab and the association of those antibodies with SIRs. The 545 patients consisted of 155 case–control patients and an additional 390 patients who were not matched to cases. Twenty patients with case–controls met the protocol-defined criteria of SIR and were included in the analysis; one additional patient with an SIR had no matching control available and was not included in the assessment of the primary objective. The remaining 524 patients did not meet the criteria for SIRs.

The demographics of all 545 patients and the subset of 155 case–control patients are summarized in Table S1. The study population included patients with colorectal, head and neck, or lung cancer. Characteristics were generally similar in all patients versus the case–control subgroup and across those who experienced an SIR versus those who did not. Due to the small number of samples available from each location and the retrospective nature of the data, the correlation between the geographic location and the incidence of SIR could not be assessed. The frequency and percentage of patients who experienced an SIR by site are shown in Table S2.

### IgE cross-reactivity and association with SIRs

In the subset of patients with site-matched controls, 58% of patients who tested positive for IgE cross-reactive with cetuximab experienced an SIR (PPV), whereas 96% of those who did not test positive did not experience an SIR (NPV) (Table2). The odds of a patient who tested positive for the presence of cetuximab IgE antibodies having an SIR was 62 times that of a patient who tested negative (*P* ≤ 0.0001) (Table2). The sensitivity of the assay was 0.750 (95% CI, 0.509–0.913), whereas the specificity was 0.919 (95% CI, 0.859–0.959).

**Table 2:** Association of IgE status with severe HSRs: odds ratio and predictive value of HSR reaction.

	Patients with site-matched controls (*N* = 155)		
	Severe HSR (*n* = 20)	No severe HSR (*n* = 135)	Predictive value
IgE positive	15	11	Positive: 0.577 (0.369–0.766)
IgE negative	5	124	Negative: 0.961 (0.912–0.987)
Odds ratio^1^	61.994 (7.637–503.25) *P* ≤ 0.0001		
Sensitivity	0.750 (0.509–0.913)		
Specificity	0.919 (0.859–0.959)		

	All patients (*N* = 545)		
	Severe HSR (*n* = 21)	No severe HSR (*n* = 524)	Predictive value
IgE positive	15	23	Positive: 0.395 (0.240–0.566)
IgE negative	6	501	Negative: 0.988 (0.974–0.996)
Sensitivity	0.714 (0.478–0.887)		
Specificity	0.956 (0.935–0.972)		

HSR, hypersensitivity reaction.

Odds of a patient testing positive to IgE having a severe HSR compared with the odds of a patient testing negative for IgE having a severe HSR.

For all patients with IgE measurements combined, 99% of the patients with samples who tested negative for IgE antibodies cross-reactive with cetuximab did not experience a severe HSR (NPV), and 40% of the patients who tested positive for IgE antibodies cross-reactive with cetuximab experienced an SIR (PPV) (Table2). The sensitivity of the assay in all patients with IgE measurements was 0.714 (95% CI, 0.478–0.887), and its specificity was 0.956 (95% CI, 0.935–0.972).

There were both false-positive and false-negative results (Table2). In the subset of patients with matched controls, 11 patients tested IgE positive but had no reference in their clinical records of having experienced an SIR that met the study criteria (false-positive results) and five patients had a documented SIR but had a negative IgE test result (false-negative results). In the all-patient group, 23 had false-positive results, whereas six patients had false-negative results.

An exploratory analysis (Table3) suggested that the majority of patients (14 of 21) who experienced an SIR had titers of anti-cetuximab IgE ≥0.35 kU_A_/L, and the majority of patients (14 of 21) with titers ≥0.35 kU_A_/L experienced an SIR.

**Table 3:** IgE antibodies to cetuximab as predictors of hypersensitivity reactions during the first infusion of cetuximab in all patients (*n* = 545)—exploratory analysis of different cutoffs.

	Titer of IgE antibodies to cetuximab		
	≤0.10 kU_A_/L *n* = 507	>0.10 to <0.35 kU_A_/L *n* = 17	≥0.35 kU_A_/L *n* = 21
SIR	6	1	14
No SIR	501	16	7

SIR, severe infusion reaction.

## Discussion

Biological agents, including cetuximab, are associated with varying frequencies of mild to life-threatening infusion-related reactions 1–6. SIRs occur in 2–5% of patients treated with cetuximab. Preexisting IgE antibodies cross-reacting with a glycosylation site in the Fab fragments of cetuximab were previously found to be associated with SIRs 11. A goal of the current study was to evaluate this association in a larger independent population.

The study included patients with various tumor types who received various lines of treatment and excluded patients analyzed by Chung et al. 11. The overall incidence of SIRs in the study population was ∼4%, consistent with the frequency for cetuximab in the U.S. 5. Thus, this population appears to be representative of a typical cetuximab-treated population. Nevertheless, it represents a convenience sample, because the patients were included retrospectively based on the availability of a blood sample and appropriate consent.

In the case–control sample set, pretreatment IgE antibodies cross-reactive with cetuximab were correlated with the occurrence of SIRs during initial infusion. Patients with a positive test result had a 62-fold higher risk of experiencing an SIR, supporting previous observations 11. However, the presence of IgE antibodies did not result in SIRs in the majority of patients. Conversely, nearly one-third (six of 21) of patients who experienced an SIR did not have preexisting IgE; thus, the absence of specific IgE does not preclude infusion reactions with cetuximab, which may be mediated by other mechanisms.

A predictive marker may gain clinical utility if test-positive patients can be treated and/or managed differently compared with test-negative patients. In our study, test-negative patients had a high likelihood of not developing an SIR (98.8%; 95% CI, 97.4–99.6). However, physicians treating these patients would still need to follow the same precautions recommended for cetuximab administration. Alternatively, test-positive patients had a 39.5% (95% CI, 24.0–56.6) risk of experiencing an SIR, for example, the presence of IgE antibodies >0.1 did not result in SIRs in 23 of 38 patients. Furthermore, as reflected by broad CIs, the true risk in test-positive patients is difficult to estimate due to the moderate sample size of this study and the (expected) low incidence of SIRs. Therefore, each patient must be evaluated carefully for risk versus benefit before receiving cetuximab, regardless of the test results.

The PPV of a test can also be increased by limiting testing to a population with a higher baseline risk. A higher incidence of SIRs in some areas in the southeastern U.S. has been observed 8,11, and it has been suggested that the lone star tick prevalent in this area may contribute to the development of anti-alpha-gal IgE 12. Importantly, our study included patients from a broad range of sites across the U.S. Although most patients testing positive for IgE antibodies were treated in the southeastern U.S., SIRs and anti-cetuximab IgE were observed elsewhere, and establishing a definitive association between location and the presence of anti-cetuximab IgE or risk of SIR may be difficult to determine in a mobile society. Of note, in a similar retrospective study, anti-cetuximab IgE was identified in a similar proportion in patients in France 16. Thus, the risk of experiencing an SIR or the existence of pretreatment IgE cross-reactive with cetuximab may not be limited to the Southeast area of the United States.

This large validation study provides substantial evidence of an association of cross-reacting IgE antibodies and an increased risk of SIRs. The main limitations of the study include its retrospective nature, the use of a convenience sample, and the expected small number of patients with an infusion reaction. Due to the retrospective nature of the study, relevant clinical information regarding the HSRs may have been missing in the case report forms. Because the frequency of SIRs was low (as expected), the data were associated with wide CIs, which made it difficult to assess the true strength of the association. The analyses may be further complicated by the likelihood of multiple underlying mechanisms for HSRs.

It is currently unclear whether the mere presence of IgE antibodies predisposes individuals to a severe HSR upon exposure or whether a certain threshold is required. This manuscript provides the first evidence that the detection limit may affect the utility of an assay designed to measure the presence or absence of anti-cetuximab IgE. The earlier study investigating a potential correlation between anti-cetuximab IgE and severe HSR used an assay with a detection limit of 0.35 kU_A_/L, whereas the assay used in the current study has a detection limit of 0.10 kU_A_/L. Our exploratory analysis using the ≥0.35 kU_A_/L cutoff suggests that the degree of association between the presence of IgE and the likelihood of SIRs occurring will depend on the assay cutoff. While these data should be considered exploratory and hypothesis generating, they provide valuable information about the complexities of designing diagnostic assays based on the retrospectively obtained samples.

## Supporting Information

**Table S1.** Patient demographics and baseline characteristics.

**Table S2.** The frequency and percentage of subjects that experience, or do not experience a severe HSR by geographic site.
