# Supplementary material for: A retrospective analysis of cross-reacting cetuximab IgE antibody and its association with severe infusion reactions
Source: Cancer Med. 2014 Oct 9;4(1):36–42. doi: 10.1002/cam4.333 (PMC4312116; doi:10.1002/cam4.333)
Supplement: Supplementary file 1 [file cam40004-0036-sd1.docx]

**Supporting Information**

**Table 1.** Patient Demographics and Baseline Characteristics

| **Characteristics** | **Treated Patients**  **n = 545** | | **Case-Control Patients**  **n = 155** | |
| --- | --- | --- | --- | --- |
|  | **Severe HSR**  **n = 21** | **No Severe HSR**  **n = 524** | **Severe HSR**  **n = 20** | **No Severe HSR**  **n = 135** |
| Median age (range), y    Age range, %  21-45 y  46-65 y  66-75 y  > 75 y  Not reported | 58 (43-72)  5  67  19  0  9 | 59 (22-85)  11  46  23  5  15 | 57.5 (43-72)  5  70  15  0  10 | 58 (22-83)  13  47  27  6  7 |
| Sex, %  Male  Female | 76  24 | 56  44 | 75  25 | 55  45 |
| Race, %  White  Black  Hispanic  Asian  Other | 90  5  0  0  5 | 88  6  1  3  2 | 90  5  0  0  5 | 90  7  0  1  2 |
| Tumor type, %  Breast  Colorectal  Head and neck  Lung  Ovarian  Pancreatic  Other | 0  52  5  28  10  0  5 | 1  81  6  6  1  2  3 | 0  50  5  30  10  0  5 | 4  64  10  13  4  0  5 |
