# Supplementary material for: A retrospective analysis of cross-reacting cetuximab IgE antibody and its association with severe infusion reactions
Source: Cancer Med. 2014 Oct 9;4(1):36–42. doi: 10.1002/cam4.333 (PMC4312116; doi:10.1002/cam4.333)
Supplement: Supplementary file 2 [file cam40004-0036-sd2.docx]

| **Table S.6B:** | | |
| --- | --- | --- |
| **The Frequency and Percentage of Subjects that Experience, or do Not Experience a Severe HSR by Geographic Site** | | |
| **Data set: All Treated Subjects with IgE Measurements** | | |
|  |  |  |
|  | Subjects that Experienced a Severe HSR (%) (1) | Subjects that did Not Experience a Severe HSR (%) (1) |
| Arlington Cancer Center |  | 6 ( 100.00 ) |
| Bendheim Cancer Center |  | 13 ( 100.00 ) |
| Beth Israel Medical Center |  | 1 ( 100.00 ) |
| California Cancer Care |  | 2 ( 100.00 ) |
| Cancer Care Specialists | 1 ( 10.00 ) | 9 ( 90.00 ) |
| Cancer Center of Boston |  | 4 ( 100.00 ) |
| Cancer Institute Medical Group |  | 3 ( 100.00 ) |
| Cancer Institute of New Jersey | 1 ( 5.00 ) | 19 ( 95.00 ) |
| Caritas St. Elizabeth's Medical Center |  | 1 ( 100.00 ) |
| Carolinas Hematology-Oncology Associates |  | 2 ( 100.00 ) |
| Center for Cancer Care |  | 10 ( 100.00 ) |
| Center for Hem/Onc |  | 1 ( 100.00 ) |
| Center for Oncology Research |  | 16 ( 100.00 ) |
| City of Hope Medical Center |  | 12 ( 100.00 ) |
| Cliniques Universitaires Saint Luc |  | 7 ( 100.00 ) |
| Commonweatlth Cancer Center |  | 2 ( 100.00 ) |
| Cooper Cancer Institute |  | 6 ( 100.00 ) |
| Dana-Farber Cancer Institute | 2 ( 9.09 ) | 20 ( 90.91 ) |
| Division of Hematology/Oncology University of North Carolina | 1 ( 10.00 ) | 9 ( 90.00 ) |
| Duke University Medical Center |  | 2 ( 100.00 ) |
| Dunedin Hospital |  | 1 ( 100.00 ) |
| East Jefferson Specialty Cntr |  | 2 ( 100.00 ) |
| Florida Cancer Specialist | 1 ( 16.67 ) | 5 ( 83.33 ) |
| Florida Hospital |  | 11 ( 100.00 ) |
| Fox Chase Cancer Center |  | 5 ( 100.00 ) |
| Georgetown Univ Medical Center |  | 5 ( 100.00 ) |
| Georgia Cancer Specialist |  | 1 ( 100.00 ) |
| Greater Baltimore Medical Ctr |  | 3 ( 100.00 ) |
| Greenwich Hospital |  | 6 ( 100.00 ) |
| Gynecologic Oncology | 1 ( 25.00 ) | 3 ( 75.00 ) |
| Hanover Medical Specialists, Pa | 1 ( 50.00 ) | 1 ( 50.00 ) |
| Hem/Onc Assoc of Illinois |  | 4 ( 100.00 ) |
| Hem/Onc Centers of Northern Rockies |  | 2 ( 100.00 ) |
| Highlands Oncology Group | 1 ( 50.00 ) | 1 ( 50.00 ) |
| Hollen, Charles W |  | 1 ( 100.00 ) |
| Hospital General Vall d"Hebron |  | 2 ( 100.00 ) |
| Hospital Virgen de la Arrixaca |  | 1 ( 100.00 ) |
| Indiana Cancer Pavilion |  | 8 ( 100.00 ) |
| Indiana University |  | 6 ( 100.00 ) |
| Institute Jules Bordet | 1 ( 16.67 ) | 5 ( 83.33 ) |
| Integrated Community Oncology Network |  | 1 ( 100.00 ) |
| Intermountain Hem/Onc Assoc |  | 1 ( 100.00 ) |
| Jefferson Medical College | 1 ( 20.00 ) | 4 ( 80.00 ) |
| Kaiser Permanente Medical Center |  | 1 ( 100.00 ) |
| Lakeland Regional Cancer Center |  | 14 ( 100.00 ) |
| Lee Moffitt Cancer Center |  | 12 ( 100.00 ) |
| MD Anderson Cancer Center |  | 7 ( 100.00 ) |
| Mayo Clinic Jacksonville |  | 1 ( 100.00 ) |
| Medical Oncology L.L.C. |  | 2 ( 100.00 ) |
| Memorial Sloan-Kettering |  | 30 ( 100.00 ) |
| Memphis Cancer Center |  | 13 ( 100.00 ) |
| Miami VA Medical Center |  | 1 ( 100.00 ) |
| Missouri Cancer Care |  | 5 ( 100.00 ) |
| New York University | 1 ( 4.55 ) | 21 ( 95.45 ) |
| Newark Beth Israel Medical Center Division of Hematology/Onc | 1 ( 50.00 ) | 1 ( 50.00 ) |
| North Idaho Cancer Center |  | 2 ( 100.00 ) |
| North Shore Hema/Oncology |  | 9 ( 100.00 ) |
| North Texas Regional Cancer Center |  | 1 ( 100.00 ) |
| Northern Utah Associates |  | 1 ( 100.00 ) |
| Nottingham City Hospital |  | 1 ( 100.00 ) |
| Ocshner Clinical Foundation |  | 1 ( 100.00 ) |
| Onc/Hem grp of South Florida |  | 2 ( 100.00 ) |
| Oncology & Hematology (Metairie,LA) |  | 4 ( 100.00 ) |
| Pacific Shores Medical | 1 ( 7.14 ) | 13 ( 92.86 ) |
| Park Nicollet Onc Research Program |  | 1 ( 100.00 ) |
| Peachtree Hem/Onc | 1 ( 11.11 ) | 8 ( 88.89 ) |
| Pennsylvania Onc/Hem Assoc |  | 4 ( 100.00 ) |
| Piedmont Hematology Oncology | 1 ( 50.00 ) | 1 ( 50.00 ) |
| Radiation Oncology Center |  | 2 ( 100.00 ) |
| Rocky Mountain Cancer Center |  | 14 ( 100.00 ) |
| Saleh, Mansoor |  | 1 ( 100.00 ) |
| Sarah Cannon Cancer Center | 3 ( 33.33 ) | 6 ( 66.67 ) |
| Scott & White Hospital |  | 2 ( 100.00 ) |
| Sidney Kimmel Comp CA Ctr @ John Hopkins |  | 3 ( 100.00 ) |
| St. Joseph Mercy Hospital |  | 5 ( 100.00 ) |
| Stockton Hem./Onc. Med. Group |  | 1 ( 100.00 ) |
| Swedish Cancer Institute |  | 14 ( 100.00 ) |
| Tower Hem/Onc Cancer Research |  | 6 ( 100.00 ) |
| Trident Medical Center |  | 1 ( 100.00 ) |
| Trident Palmetto Hema/Oncology |  | 1 ( 100.00 ) |
| UCLA Medical Center |  | 2 ( 100.00 ) |
| USC Norris Cancer Center |  | 44 ( 100.00 ) |
| University Hosp Gasthuisberg |  | 23 ( 100.00 ) |
| University of Alabama |  | 3 ( 100.00 ) |
| University of Chicago |  | 3 ( 100.00 ) |
| University of Colorado |  | 1 ( 100.00 ) |
| University of Virginia | 1 ( 10.00 ) | 9 ( 90.00 ) |
| University of the Free State |  | 2 ( 100.00 ) |
| Yale University | 1 ( 100.00 ) |  |
| Yanagihara, Ronald H., MD |  | 1 ( 100.00 ) |
|  |  | Total = 545 |

(1) Based on the CA225-317 HSR criteria , described in the charter document.
